# Neutrophil elastase decreases SARS-CoV-2 spike protein binding to human bronchial epithelia by clipping ACE-2 ectodomain from the epithelial surface

**DOI:** 10.1016/j.jbc.2023.104820

**Published:** 2023-05-13

**Authors:** Apparao B. Kummarapurugu, Adam M. Hawkridge, Jonathan Ma, Stephanie Osei, Rebecca K. Martin, Shuo Zheng, Judith A. Voynow

**Affiliations:** 1 Department of Pediatric Pulmonary Medicine, Children’s Hospital of Richmond, Virginia Commonwealth University, Richmond, Virginia, USA; 2 School of Pharmacy, Virginia Commonwealth University, Richmond, Virginia, USA; 3 Virginia Commonwealth University, Richmond, Virginia, USA; 4 Department of Microbiology and Immunology, Virginia Commonwealth University, Richmond, Virginia, USA

**Keywords:** neutrophil, proteinase, airway epithelial cell, virus entry, spike protein, ectodomain cleavage, mass spectrometry, SARS-CoV-2, ACE-2

## Abstract

Patients with cystic fibrosis (CF) have decreased severity of severe acute respiratory syndrome-like coronavirus-2 (SARS-CoV-2) infections, but the underlying cause is unknown. Patients with CF have high levels of neutrophil elastase (NE) in the airway. We examined whether respiratory epithelial angiotensin-converting enzyme 2 (ACE-2), the receptor for the SARS-CoV-2 spike protein, is a proteolytic target of NE. Soluble ACE-2 levels were quantified by ELISA in airway secretions and serum from patients with and without CF, the association between soluble ACE-2 and NE activity levels was evaluated in CF sputum. We determined that NE activity was directly correlated with increased ACE-2 in CF sputum. Additionally, primary human bronchial epithelial (HBE) cells, exposed to NE or control vehicle, were evaluated by Western analysis for the release of cleaved ACE-2 ectodomain fragment into conditioned media, flow cytometry for the loss of cell surface ACE-2, its impact on SARS-CoV-2 spike protein binding. We found that NE treatment released ACE-2 ectodomain fragment from HBE and decreased spike protein binding to HBE. Furthermore, we performed NE treatment of recombinant ACE-2-Fc–tagged protein *in vitro* to assess whether NE was sufficient to cleave recombinant ACE-2-Fc protein. Proteomic analysis identified specific NE cleavage sites in the ACE-2 ectodomain that would result in loss of the putative N-terminal spike-binding domain. Collectively, data support that NE plays a disruptive role in SARS-CoV-2 infection by catalyzing ACE-2 ectodomain shedding from the airway epithelia. This mechanism may reduce SARS-CoV-2 virus binding to respiratory epithelial cells and decrease the severity of COVID19 infection.

Cystic fibrosis (CF) is a life-threatening multisystem disease caused by mutations in the CF transmembrane conductance regulator gene. It is characterized by neutrophil-driven inflammation with gradually worsening bronchiectasis causing pulmonary insufficiency, the main cause of mortality (1). Neutrophil elastase (NE), present in high concentrations in CF airway surface fluids, impairs airway innate immune mechanisms and promotes inflammation (2). Patients with CF are highly vulnerable to viral infections not only due to their chronic lung disease but also because of impaired antiviral innate immunity (3, 4).

The novel severe acute respiratory syndrome-like coronavirus-2 (SARS-CoV-2) is responsible for the COVID-19 global pandemic and has caused significant morbidity and mortality (5). SARS-CoV-2 uses the angiotensin-converting enzyme 2 (ACE-2) as a host cellular receptor to initiate infection (6, 7, 8, 9, 10) in respiratory epithelia, the point of initial viral entry. ACE-2 protein, localized to the apical domain of the plasma membrane of epithelial cells, is a transmembrane glycoprotein consisting of a short C-terminal cytoplasmic tail, a hydrophobic transmembrane domain, and a heavily N-glycosylated N-terminal ectodomain (11). It is highly expressed in the nasal epithelia, the large airway epithelia, and alveolar type 1 and 2 epithelia (12). Interestingly, CF epithelia have greater abundance of ACE-2 expression than non-CF airway epithelia (12). However, fewer patients with CF are infected with SARS-CoV-2 and those that are infected appear to have better outcomes than initially anticipated, with their COVID-19 disease following a course similar to or better than the general population (13, 14, 15).

The reasons for less severe COVID-19 infections in patients with CF are perplexing given their susceptibility to other respiratory infections and higher levels of ACE-2 expression. This may be due to disease prevention strategies with increased vaccination rates, mask wearing, and social distancing. However, there may be physiologic reasons for less severe disease in patients with CF (16). The transmembrane serine protease 2 mRNA levels may be decreased in CF, which would inhibit SARS-CoV-2 infection. Although increased ACE-2 mRNA in CF epithelia (12) should increase the abundance of viral receptors, it also would result in increased conversion of angiotensin 2 to angiotensin 1 to 7 which is anti-inflammatory. Many patients with CF are on chronic azithromycin therapy which is also anti-inflammatory and inhibits furin activity (16). We have been studying the effect of NE in the CF airway and considered whether NE may paradoxically interrupt the interaction between the SARS-CoV-2 spike protein and ACE-2 on respiratory epithelial cells.

Recent studies suggest that human recombinant soluble ACE-2 (sACE-2) acts as a decoy to bind SARS-CoV-2 spike protein and inhibit infection (17, 18). The ACE-2 ectodomain is released from the cell surface by a transmembrane proteinase, a disintegrin and metalloprotease17 (ADAM17) by cleavage between amino acids 716 and 741 (19). sACE-2 is also increased in sputum from patients with severe neutrophil-dominant asthma and the release is attributed to increased furin levels (20). The CF airway is rich in unopposed proteases including NE, matrix metalloproteinase-8, matrix metalloproteinase-9, cathepsin G, and lysosomal cathepsins (2), which may potentially release sACE-2, generating a decoy receptor in the airway to bind virus, reducing viral binding to the epithelial surface (21, 22). NE not only activates other proproteinases, it also cleaves and/or regulates expression of many proteins in the airway (23). We hypothesized that high levels of NE in sputum of patients with CF may cleave and release the ACE-2 ectodomain from respiratory epithelial cells. One consequence of this cleavage would be to decrease the ACE-2 availability at the epithelial surface for SARS-CoV-2 spike binding and viral infection.

## Results

### sACE-2 levels were increased in sputum but not in plasma in patients with CF compared to non-CF subjects

**Figure 1:**
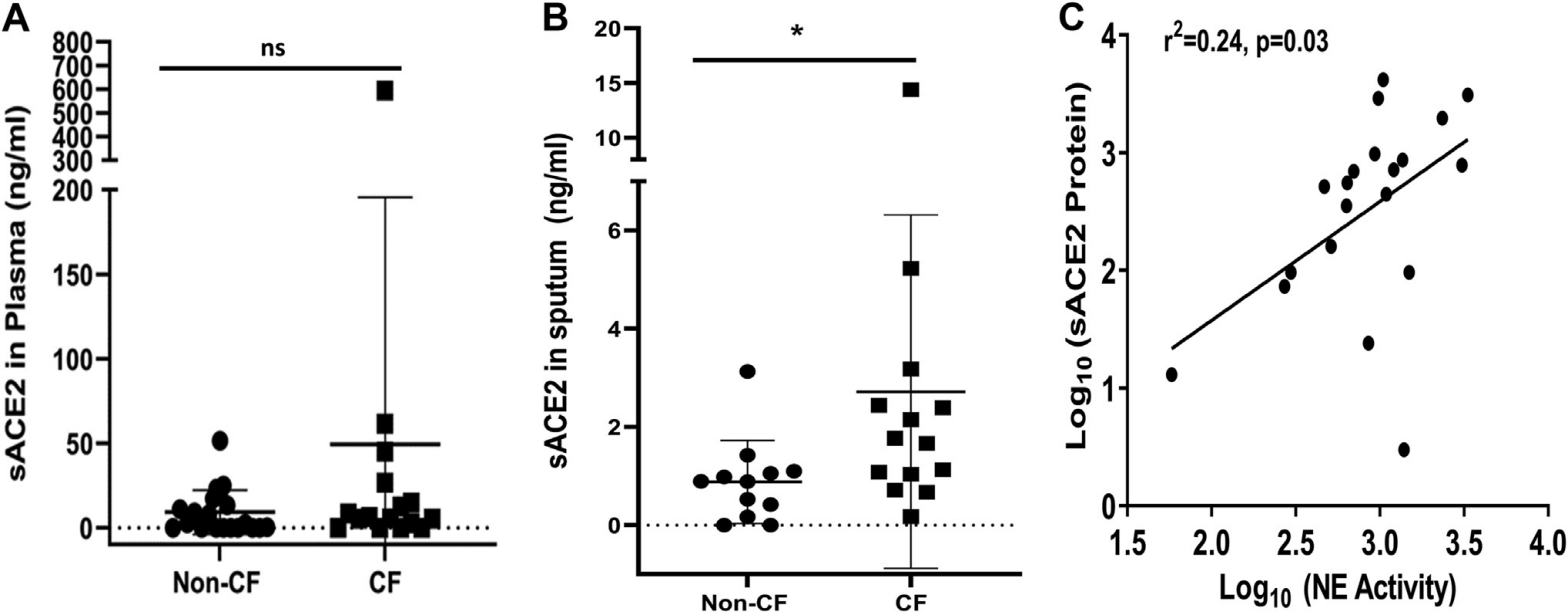
**Quantitation of ACE-2 levels in plasma and airway mucus from subjects with and without CF and association of ACE-2 levels in CF sputum with neutrophil elastase activity.** Blood samples collected from subjects with or without CF were processed for plasma collection, aliquoted, and stored at −80 °C until further use. Frozen sputa from patients with CF or mucus from endotracheal tubes (ETTs) of healthy adult patients were mixed with NS containing 10% Sputolysin at 1:1 (sputum/mucus [mg]: volume [μl]) (37 °C, 15 min), and sputum/mucus supernatants were collected by centrifugation. Plasma and sputum and mucus supernatants were analyzed for sACE-2 by ELISA. Data are summarized as (mean ± SEM) for plasma samples (20 non-CF and 16 CF) (*A*) and sputum/mucus samples (12 non-CF and 14 CF) (*B*). Statistical comparisons were made using Mann–Whitney U test. Soluble ACE-2 levels were significantly increased in CF sputum compared to non-CF, ∗*p* = 0.0146. For analysis of sputum NE activity compared to sputum ACE-2 levels, sputum supernatants prepared with DNase-1 (0.3 mg/ml) for 2 h at 37 °C were used. Scatter *dot plot* showed a linear correlation between ACE-2 and NE activity levels in CF sputum supernatants (n = 20, r^2^ = 0.24, *p* = 0.03) (*C*). ACE-2, angiotensin-converting enzyme 2; CF, cystic fibrosis; NE, neutrophil elastase; NS, normal saline; sACE-2, soluble ACE-2.

We first sought to determine whether ACE-2 protein levels were increased in the CF airway due to high abundance of NE serine proteinase activity that either directly or indirectly catalyzes the release of the ACE-2 ectodomain. sACE-2 levels were quantified by ELISA in CF plasma and sputum and in non-CF plasma and non-CF tracheal mucus as control bio specimens. CF sputum (Fig. 1*B*) but not plasma (Fig. 1*A*) had significantly increased sACE-2 levels compared to non-CF bio specimens. It should be noted that the number of samples, 12 non-CF and 14 CF, were sufficient to show statistically significant increase in sACE-2 protein in CF sputum compared to non-CF tracheal mucus. In addition, there was a linear correlation between NE activity levels and sACE-2 protein abundance in CF sputum (Fig. 1*C*) (*r*^2^ = 0.24; *p* = 0.03).

### NE mediated the release of ACE-2 fragment, the ectodomain of the ACE-2 which was detected in conditioned media

**Figure 2:**
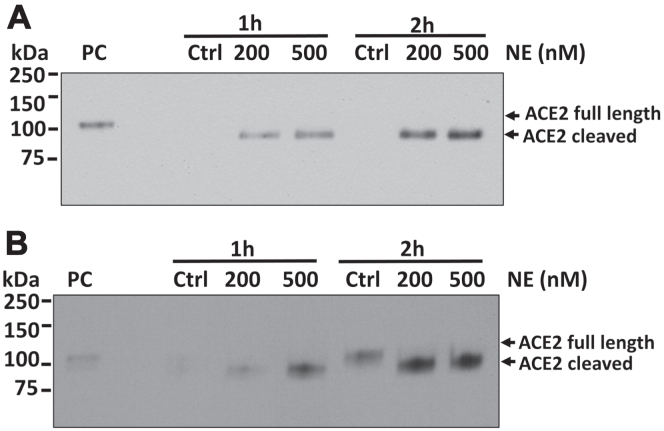
**NE treatment of HBE and release of ACE-2 ectodomain fragment in conditioned media.** HBE cells cultured on plastic (*A*) or at air–liquid interface (*B*) were treated with vehicle control or NE (200 or 500 nM) for 1 h or 2 h. Conditioned media (CM) was collected and concentrated to enrich ACE-2 ectodomain fragment release. Concentrated media (22 μl) proteins were separated on a 4 to 20% SDS-PAGE and probed for ACE-2 fragment following transfer to nitrocellulose. Blots were probed with primary rabbit anti-ACE-2 antibody that recognizes the N-terminal ACE-2 ectodomain of the protein (1:1000 dilution, O/N in 5% milk); secondary horseradish peroxidase (HRP)-conjugated goat anti-rabbit IgG antibody (1:5000 dilution) and the immunoreactive protein complexes were detected using lightning ultrachemiluminescence substrate. *A*, immunoblot showing the release of ACE-2 fragment in the conditioned media from undifferentiated NHBE cells cultured on plastic. *B*, immunoblot of apical conditioned media from differentiated NHBE cells cultured at ALI. Positive control (PC) was Caco2 whole cell lysates (catalog number ab3950, Abcam). Western blots shown were representative of n = 4 undifferentiated and n = 2 differentiated NHBE donor cells. ACE-2, angiotensin-converting enzyme 2; IgG, immunoglobulin G; HBE, human bronchial epithelial; NE, neutrophil elastase.

To determine whether the increase in sACE-2 protein in CF sputum is the result of NE proteolytic cleavage of endogenously expressed ACE-2 from airway epithelia, *in vitro* experiments with primary cultures of human bronchial epithelial (HBE) and NE exposure were performed. Both undifferentiated (cultured on plastic) and well-differentiated (air–liquid interface [ALI] culture) primary HBE cells were exposed to NE (200 or 500 nM for 1 h or 2 h) or control vehicle, and the conditioned media was evaluated by Western analysis using an antibody that detects the ectodomain of ACE-2. NE-cleaved ACE-2 protein was detected with an estimated molecular weight (MW) ∼85 kDa representing clipped ACE-2 ectodomain in the conditioned media obtained from both undifferentiated and well-differentiated primary HBE cells (Fig. 2, *A* and *B*). NE-induced shedding of ACE-2 in conditioned media was dose-dependent at 1 h but was similar between doses by 2 h. Although, well-differentiated HBE cell cultures were exposed to NE at both apical and basolateral chambers, only apical conditioned media had ACE-2 present (data not shown). Western analysis of control vehicle–treated HBE revealed low abundance, but detectable ACE-2 was released into apical conditioned media from well-differentiated HBE at 2 h incubation with MW of ∼110 kDa (Fig. 2*B*), but no detectable ACE-2 was released into conditioned media from undifferentiated HBE cells (Fig. 2*A*). These observations are consistent with NE or a NE-regulated protease catalyzing the clipping and release of the ACE-2 ectodomain into the CF airway.

### NE caused the loss of the ACE-2 from the HBE cell surface

Flow cytometry was used to evaluate whether ACE-2 ectodomain shedding following NE treatment resulted in decreased ACE-2 expression on HBE cell surface. The gating strategy for this method is shown in Supporting information (Fig. S1). Undifferentiated HBE cells, harvested using an enzyme-free cell dissociation buffer, were exposed to NE (200 or 500 nM) or vehicle control in suspension culture for 2 h and stained with goat anti-ACE-2 antibody followed by phycoerythrin (PE)-conjugated horse anti-goat secondary antibody. Mean fluorescence intensities (MFIs) were recorded from 50,000 live cells (4′, 6-diamidino-2-phenylindole [DAPI] negative) for each treatment condition by flow cytometry (Figs. S3 and 3). Robust ACE-2 surface expression was detected on control vehicle–treated HBE cells. However, following NE exposure, a significant reduction in the levels of ACE-2 membrane expression was observed on HBE cells at both doses tested when compared with control vehicle–treated cells. Specificity of ACE-2 positive staining was confirmed by control experiments including secondary antibody treatment alone or no primary and secondary antibody treatment.

### NE exposure decreased spike protein binding in HBE cells

**Figure 3:**
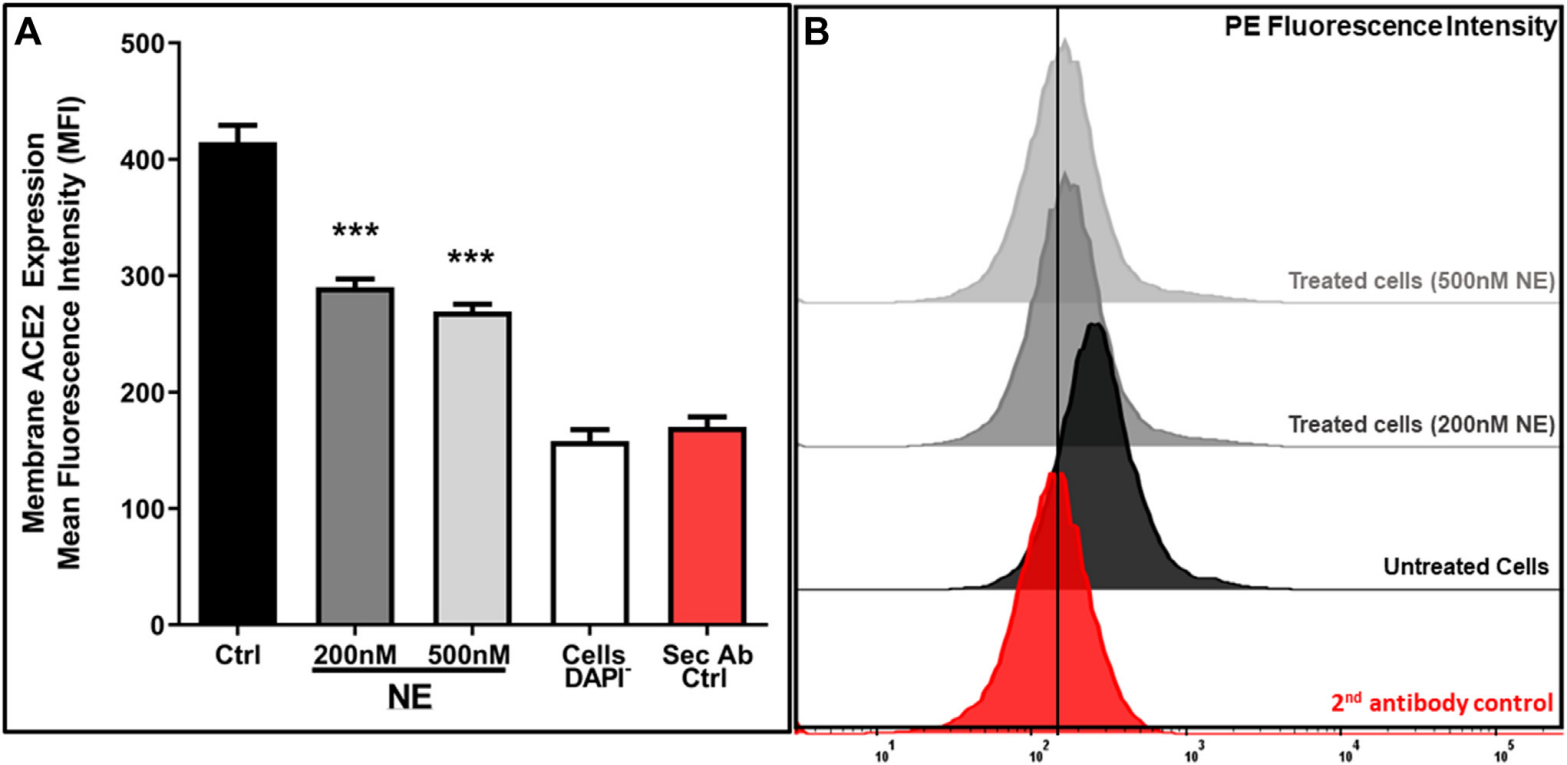
**HBE cell surface ACE-2 expression was determined by flow cytometry, following treatment with NE or control vehicle.** Cell surface ACE-2 expression in HBE cells was analyzed by flow cytometry after the cells were exposed to NE at the indicated dose. Undifferentiated HBE cells–expressing endogenous ACE-2 were exposed to NE (200 or 500 nM) or vehicle control at 37 °C for 2 h. Following incubation, the cells were stained with goat anti-ACE-2 antibody (1 μg/ml) followed by phycoerythrin-conjugated donkey anti-goat IgG (1:100). Mean fluorescence intensity (MFI) showing ACE-2 staining in HBE cells was significantly reduced with NE exposure both at 200 and 500 nM compared to untreated cells (*A*). Background MFI was determined for HBE cells exposed to no primary and no secondary (*white bar*) or secondary antibody only control (*red bar*). Representative flow cytometry histograms showing differences in ACE-2 binding to HBE cells with or without NE exposure (color matched) are shown (*B*). Data are presented as MFI ± SEM; n = 3 independent experiments with three different HBE donor cells, with a total of nine replicates per condition. ∗∗∗*p* = 0.0004 *versus*. control. Statistical analysis was performed by one-way, nonparametric ANOVA (Kruskal–Wallis test) and post hoc comparisons by Wilcoxon Rank Sum test. ACE-2, angiotensin-converting enzyme 2; HBE, human bronchial epithelial; IgG, immunoglobulin G; NE, neutrophil elastase.

**Figure 4:**
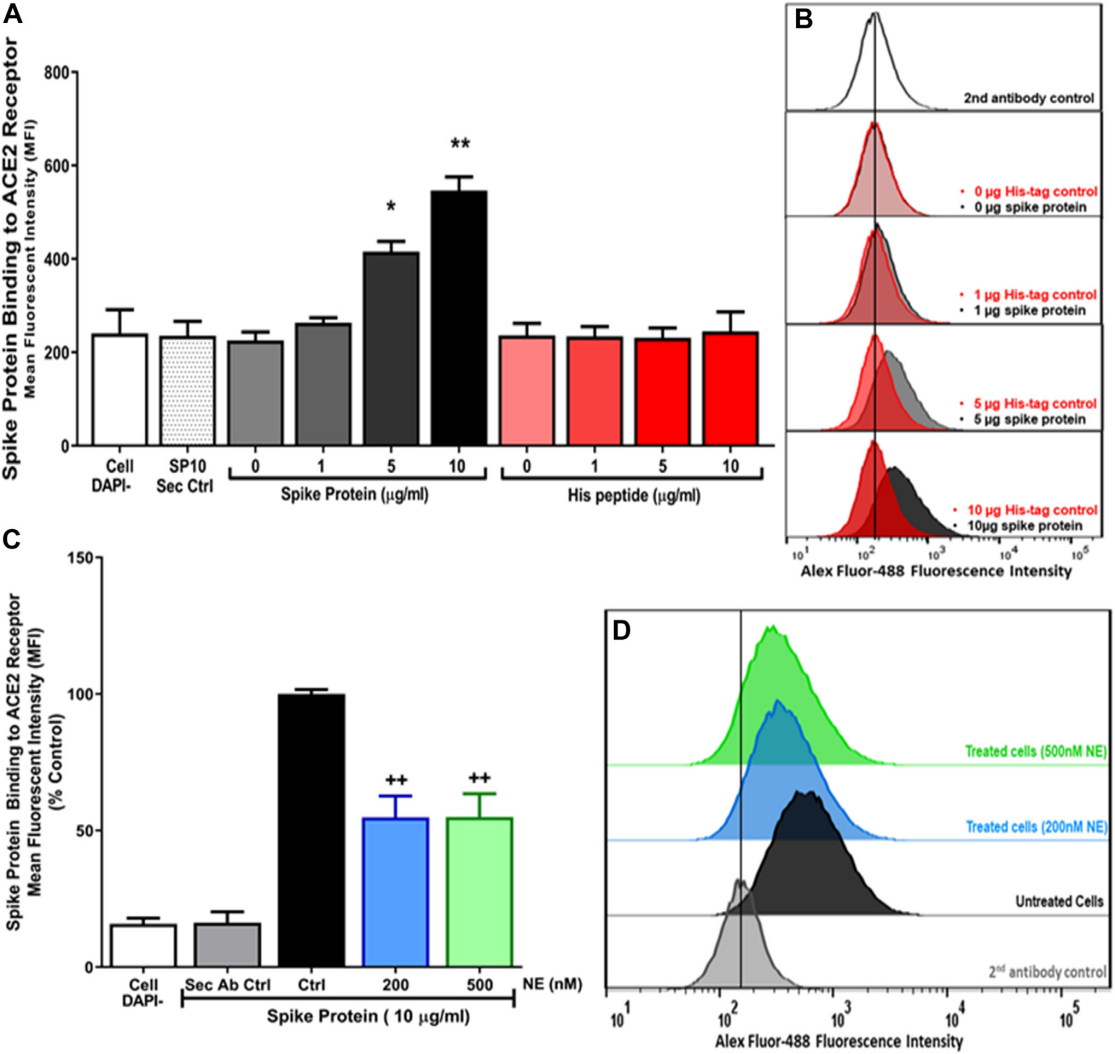
**The effect of NE treatment of HBE on SARS-CoV-2 spike protein binding detected by flow cytometry.** Dose-dependent binding of spike protein to HBE cells was determined by flow cytometry (*A* and *B*). Undifferentiated HBE cells–expressing endogenous ACE-2 were incubated with a concentration curve of recombinant SARS-CoV-2 trimeric spike protein with C-terminal His6-tag (1–10 μg/ml) or a concentration curve of His6 peptide alone as control (1–10 μg/ml), for 2 h at 37 °C. In a separate experiment, HBE were treated with NE (200 or 500 nM) or control vehicle for 2 h prior to incubation with SARS-CoV-2 spike protein (10 μg/ml) (*C* and *D*). Following incubation, the cells were stained with rabbit anti-His-tag antibody (1:500 dilution), followed by Alexa Fluor 488–conjugated goat anti-rabbit IgG (1:500). Mean fluorescence intensity (MFI) shows spike protein binding to HBE in a dose-dependent manner (*A*) *black bars*; however, there was only background binding with no dose-dependent increase in control His6 peptide binding to HBE (*A*) (*red bars*). Representative flow cytometry histograms show differences in spike protein binding (*black*) and His tag binding (*red*) (*B*). NE treatment significantly decreased spike protein binding to HBE (*C*). Representative histogram overlay displayed the inhibition of spike binding to HBE post-NE exposure (*D*). Data are presented as MFI ± SEM; n = 3 independent experiments with three different donor cells, with total eight replicates per condition. ∗*p* = 0.02, ∗∗*p* = 0.01 *versus* no spike protein (0); ++*p* = 0.002 *versus* Ctrl. Statistical analysis was performed by one-way, nonparametric ANOVA (Kruskal–Wallis test) and post hoc comparisons by Wilcoxon Rank Sum test. ACE-2, angiotensin-converting enzyme 2; HBE, human bronchial epithelial; IgG, immunoglobulin G; NE, neutrophil elastase; SARS-CoV-2, severe acute respiratory syndrome-like coronavirus-2.

To determine the optimal concentration of recombinant spike protein required for binding assays to HBE, SARS-CoV-2 trimeric spike protein with C-terminal His6-tag (1–10 μg/ml) was incubated with undifferentiated HBE, and spike binding was detected by flow cytometry analysis using an anti-His-tag antibody (Fig. 4). Spike protein bound to HBE in a dose-dependent manner. As expected, incubating His6 peptide alone (1–10 μg/ml) with HBE resulted in undetectable binding by flow cytometry. The binding affinities of spike protein to ACE-2 were determined by an *in vitro* protein pull-down method using recombinant ACE-2 protein with Fc-tag and SARS-CoV-2 spike protein with a His6 tag (Fig. S2) and has been previously published (6). The results showed that SARS-CoV-2 binding to ACE-2 was increased in a dose-dependent manner, confirming the specificity of spike protein binding to ACE-2 of HBE cells (Fig. 4, *A* and *B*). Following NE exposure, there was a significant decrease in spike binding to HBE (approximately 50%) that did not vary with NE dose (200 nM *versus* 500 nM) (Fig. 4, *C* and *D*). Specificity of flow cytometry results were bolstered by the negative control conditions of no primary and secondary antibodies or secondary antibody only. Taken together, the results confirmed that the SARS-CoV-2 spike protein binding to HBE cells was decreased by NE exposure, and this decrease in binding was consistent with the relative decrease in cell surface ACE-2 associated with NE-induced proteolytic shedding (Fig. 3).

### *In vitro* NE proteinase activity clipped recombinant Fc-tagged ACE-2 but had no effect on SARS-CoV-2 trimeric His6-tagged spike protein

**Figure 5:**
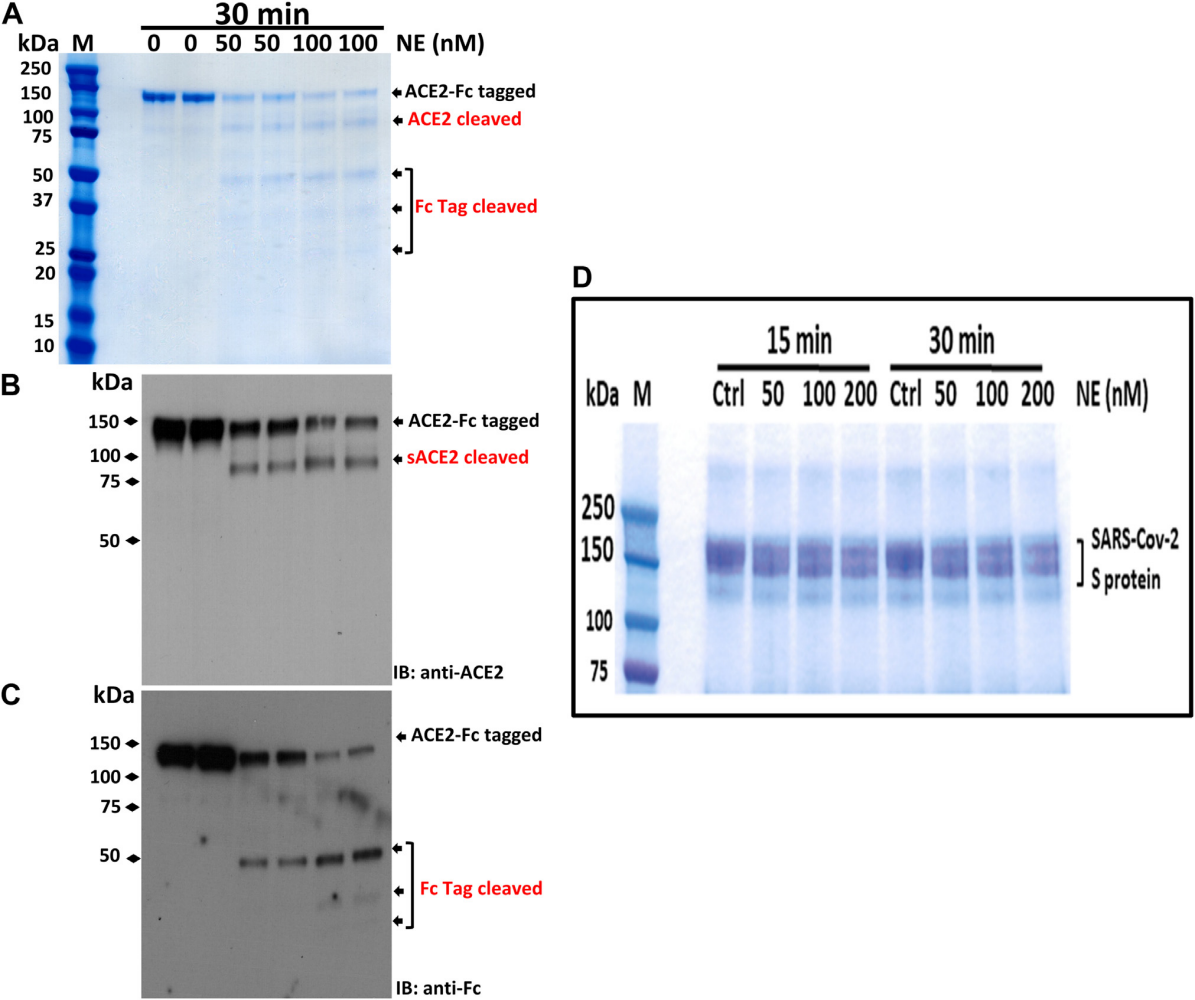
**Recombinant Fc-tagged human ACE-2 and recombinant His6-tagged SARS-CoV-2 trimeric spike protein susceptibility to NE proteinase activity.** Recombinant human ACE-2 protein with Fc-tag or SARS-CoV-2 spike protein with His6 Tag were incubated with control vehicle or NE (50, 100 nM for 15 min) for ACE2 protein or NE (50, 100, 200 nM for 15 and 30 min) for SARS-CoV-2 spike protein. Equal amounts of reaction products were resolved on 4 to 20% SDS-PAGE. ACE-2 protein tagged with Fc, cleaved by NE proteinase activity, was confirmed by imperial protein stain (*A*). The specificity of ACE-2 cleavage (*B*) or Fc cleavage (*C*) was further confirmed by probing with primary rabbit mAbs for ACE-2 (1:1000) or mouse mAb raised against human IgG_1_ Fc peptide, followed by secondary horseradish peroxidase (HRP)-conjugated goat anti-rabbit or anti-mouse IgG antibody (1:5000). Immunoreactive complexes were developed by chemiluminescence. SARS-CoV-2 spike protein was not cleaved by NE proteinase activity, confirmed by imperial protein stain (*D*). *Arrows* indicate full-length and cleaved ACE-2 or Fc fragments following NE treatment. Data shown are representative of 2 to 3 independent experiments. ACE-2, angiotensin-converting enzyme 2; IgG, immunoglobulin G; NE, neutrophil elastase; SARS-CoV-2, severe acute respiratory syndrome-like coronavirus-2.

To test whether NE proteinase activity clipped ACE-2 and/or SARS-CoV-2 spike protein, recombinant proteins of human ACE-2 with Fc-tag and SARS-CoV-2 trimeric spike protein with His6 tag were incubated with NE (50, 100, and 200 nM) for 15 or 30 min at 37 °C. Following incubation, reaction products were resolved on SDS-PAGE and visualized the cleavage pattern of Fc-ACE-2 in a dose-dependent manner (Figs. 5*A* and S4) and spike protein (Fig. 5*D*) with imperial protein stain. Western analysis for ACE-2 using an antibody specific to the ectodomain of ACE-2 following NE exposure at 30 min resulted in predominantly two bands consisting of a less intense ∼140 kDa band representing full-length human ACE-2-Fc tag with increasing dose of NE and a smaller fragment of ACE-2 at ∼85 kDa which was not present in the control vehicle–treated sample (Fig. 5*B*) and which was not present in smaller fragments detected only by anti-Fc antibody (Fig. 5*C*). The size of the smaller band ∼85 kDa represents the clipped ACE-2 ectodomain fragment released by NE proteinase activity. The Western analysis with anti-Fc antibody revealed that the protein bands appearing at ∼45, ∼35, and ∼25 kDa (Fig. 5*A*) were Fc-tag fragments. Furthermore, Western blot analysis using ACE-2 antibody did not detect in any of these low MW bands (Fig. 5*B*). Importantly, NE exposure did not cause any cleavage of His6-tagged SARS-CoV-2 trimeric spike protein under similar NE treatment conditions (Fig. 5*D*), consistent with the concept that NE-induced decrease in SARS-CoV-2 spike protein binding to HBE-associated ACE-2 was due to NE cleavage of ACE-2 and not due to NE degradation of spike protein in the airway milieu.

### Proteomic analysis of NE cleavage sites in Fc-tagged ACE-2 revealed loss of putative N-terminal spike protein-binding domain

**Figure 6:**
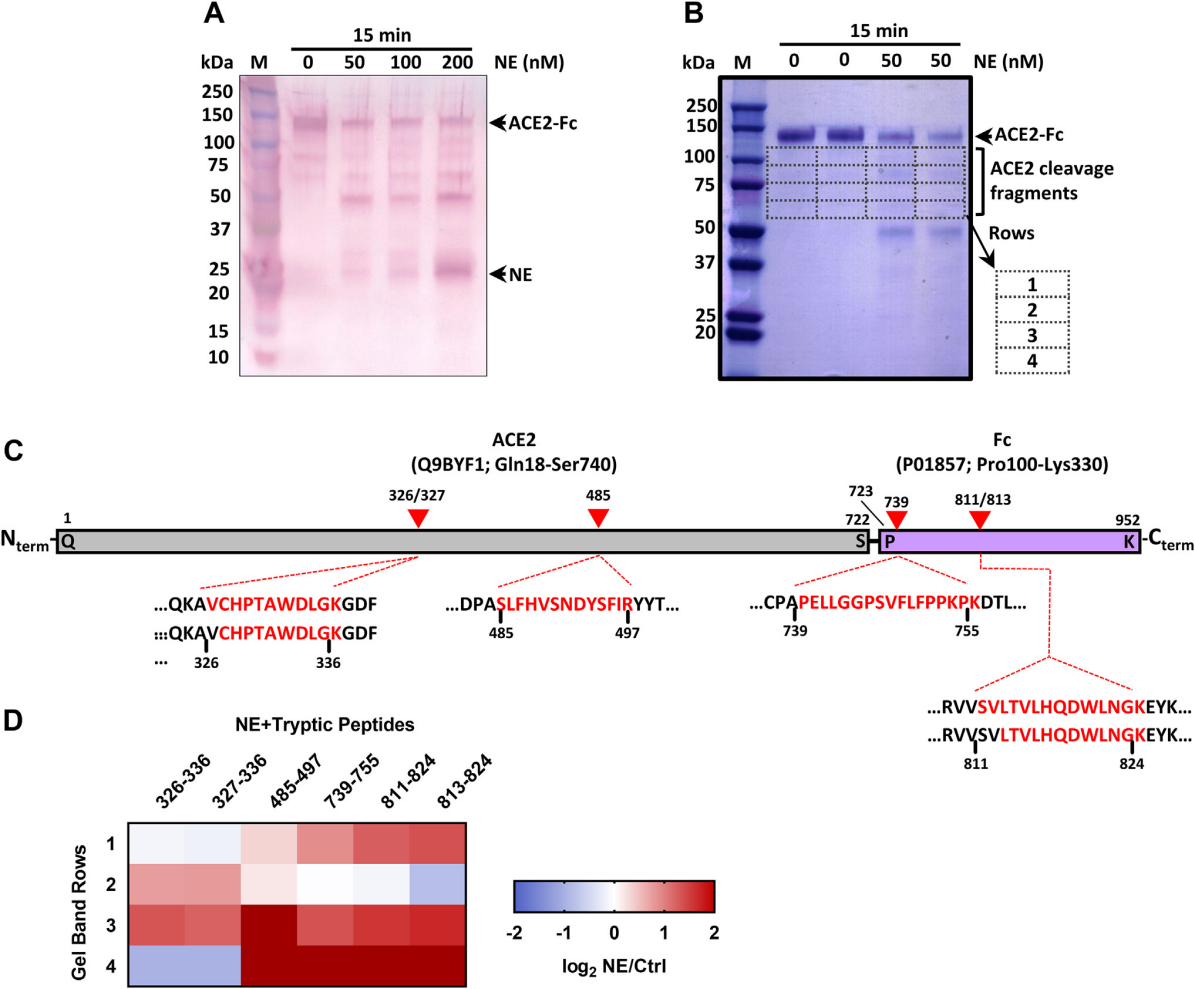
**In-gel LC-MS/MS characterization of ACE-2 cleavage sites by NE.** The ACE-2 protein with Fc-tag was treated for 15 min with neutrophil elastase at 0 (Ctrl), 50, 100, and 200 nM, separated on a 4 to 20% PAGE, and stained with highly sensitive colloidal gold total protein stain to detect full-length and NE-cleaved protein fragments (*A*). A second 4 to 20% PAGE gel was prepared under identical conditions in duplicate for ACE-2-Fc protein treated with NE at 0 (Ctrl) and 50 nM. The gel was stained with imperial protein stain, followed by excision of 16 gel fragments from four rows from regions outlined by the *dashed*-*line* grid spanning ∼120 kDa (“row 1”) to ∼65 kDa (“row 4”) and then analyzed by LC-MS/MS following in-gel tryptic digestion, (*B).* The LC-MS/MS dataset was searched using enzyme specificities for both trypsin (C-term of R, K) and neutrophil elastase (C-term of A, V) which resulted in the identification of six NE-cleavage sites including three in the ACE-2 domain (*C*). Relative label-free quantities for the six identified NE-cleaved semitryptic peptides are reported as log_2_ NE/Ctrl by gel band row (*i.e.*, MW) and amino acid positions (*D*). Data summarize one experiment with duplicate samples. ACE-2, angiotensin-converting enzyme 2; MS/MS, tandem mass spectra; MW, molecular weight; NE, neutrophil elastase.

HBE cells treated with NE resulted in the shedding of ACE-2 and reduced HBE cell binding to spike protein compared to control vehicle–treated cells. These results suggest that NE proteinase activity potentially removed the putative spike-binding domain in ACE-2 protein (24). Having established a role for NE in the regulated shedding of ACE-2 in HBE cells, we next sought to identify potential NE cleavage sites in ACE-2 protein. A sensitive colloidal gold protein stained 4 to 20% polyacrylamide gel was prepared following a NE dose-dependent treatment (Ctrl and 50–200 nM) of Fc-tagged ACE-2 for 15 min at 37 °C. Figure 6*A*). NE treatment resulted in several small bands. Although the control vehicle–treated Fc-tagged ACE-2 also had smaller faint protein bands detected by this sensitive stain, however, these peptides were not detected by anti-ACE-2 Western analysis. To generate a preparative gel for proteomic analysis, duplicate samples of Fc-tagged ACE-2 (750 ng/lane) were incubated with NE (50 nM, 15 min) or control vehicle. The proteinase digestion products were separated on a 4 to 20% PAGE, stained with Coomassie Blue (imperial protein stain), and gel slices were prepared using the grid shown for in-gel digestion of protein bands between ∼120 kDa to 65 kDa (Fig. 6*B*), followed by LC-tandem mass spectrometry (MS/MS) analysis (25). The resulting LC-MS/MS datasets were searched against ACE-2-Fc sequence and the entire human protein database using combined trypsin/NE enzyme cleavage specificity (*i.e.*, cleavage of peptides at the C terminus of R, K, A, or V) to identify semitryptic NE-cleaved peptides. Out of 116 potential cleavage sites, six NE-cleaved semitryptic peptides were identified (Fig. 6*C*). Of these sequences, three were in the ACE-2 protein domain and three were in the Fc protein. Label-free quantitative levels of the six NE-cleaved semitryptic peptides were used to calculate relative differences at each gel band row (*i.e.*, MW band) comparing NE/control peptide abundance. Focusing on the ACE-2 domain, the 326/327-336 peptides showed slight increase in the NE-treated *versus* control-treated lanes at MW 100 to 75 kDa, followed by a decrease in NE-treated bands for the lowest MW band (∼65 kDa). In contrast, the 485 to 497 peptide showed elevated levels in the NE-treated *versus* control-treated lanes, particularly with decreasing MW starting at 100 kDa (Row 2) and maxing out in rows 3 and 4 (<100 kDa). Thus, these data provide strong evidence for there being two candidate NE cleavage sites of ACE-2, but only one NE-cleaved–tryptic digest that had increased abundance in NE-treated *versus* control vehicle–treated recombinant ACE-2 at the lower MWs (Fig. 6*D*). If NE cleaved the N-terminal domain of ACE-2 at AA 485, then the putative N-terminal ACE-2 domain required for SARS-CoV-2 spike receptor binding domain binding (AA predominantly localized from glutamine 24 to arginine 372) (24) would be missing, resulting in the failure of spike protein binding to HBE treated with NE. In addition, the band between MW 100 and 75 kDa not only had increased ACE-2 peptides but also has decreased Fc-tag peptides, consistent with Western analyses of the bands at 85 kDa positive for ACE-2 and negative for Fc post-NE treatment (Fig. 5).

## Discussion

Recent reports suggest that the rate and severity of SARS-CoV-2 viral infection are lower in individuals with CF than the general population (13, 14). The lower incidence among patients with CF may be due to adopting protective measures such as social distancing, protective masks, and vaccination. However, biological protective mechanisms unique to CF patients may exist that are unknown. In this study, we explored a potential biological mechanism by which patients with CF may be protected from SARS-CoV-2 viral infection *via* NE-mediated removal of the ACE-2 ectodomain from the airway epithelia. Using primary HBE cells, a major source of ACE-2 expression in the airway, we demonstrate for the first time that NE, abundant in the CF airways, catalyzed ACE-2 cleavage by proteolytic shedding and that loss of the putative ACE-2 N-terminal spike-binding domain on the surface of HBE was associated with decreased SARS-CoV-2 spike protein binding. Furthermore, NE catalytic activity was sufficient to cleave the ectodomain of ACE-2 *in vitro*, and this finding is consistent with the observation in CF sputum that sACE-2 levels are positively correlated with NE activity. Our findings are analogous with those in a previous report that membrane-bound ACE-2 in polarized bronchial epithelium undergoes ADAM17 cleavage at the ectodomain region resulting in the release of sACE-2 (19).

The evidence of direct involvement of NE proteinase activity in ACE-2 ectodomain cleavage presented in this study led us to use LC-MS/MS to identify potential NE cleavage sites of ACE-2 protein. Two semitryptic peptides that contain NE cleavage sites at 326^Val^/327^Cys^ and 485^Ser^ of the ACE-2 N-terminal domain were identified from low MW bands in NE-treated ACE-2-Fc samples (Fig. 6*B*). These semitryptic ACE-2 peptides contain N-terminal amino acids adjacent to predicted potential NE cleavage sites. Recent studies, based on computational docking analyses, identified amino acid residue side chains (Gln^24^, Thr^27^, Asp^30^, Lys^31^, His^34^, Asp^38^, Tyr^41^, Gln^42^, Met^82^, Tyr^83^, Lys^353^, Asp^355^, and Arg^357^) on the alpha1 helix of the N-terminal region of ACE-2 ectodomain as putative domains that interact with receptor binding domain of spike protein (26, 27). Our results showed that NE cleavage of the ACE-2 ectodomain removed these putative binding residues required for SARS-CoV-2 spike protein. Similarly, ACE-2 ectodomain cleavage by ADAM17 has shown a differential cleavage pattern, between Arg^708^ and Ser^709^ amino acid residues with a synthetic ACE-2 peptide mimetic (28) and between Arg^716^ and Ile^741^ amino acid residues with glycosylated ACE-2 protein released from HBE (19), respectively. Additionally, ACE-2 was reportedly cleaved between 697 and 716 by the transmembrane serine protease 2 (29). These reports suggest ACE-2 ectodomain cleavage varies with proteases. However, it should be noted that differences in cleavage between recombinant and full-length, nonrecombinant protein may exist due to potential conformational differences between the two (28, 30).

The severity of SARS-CoV-2 viral infection correlates with ACE-2 expression levels (31). To explore mechanisms for decreased SARS-CoV-2 infection in those with CF, we evaluated and confirmed that in primary HBE cell culture, ACE-2 shedding post-NE exposure decreased cell surface ACE-2 expression, resulting in reduced viral spike protein attachment to the epithelial cell surface. These findings are further supported by a previous report showing that ACE-2 ectodomain shedding by ADAM17 is protective against SARS-CoV viral infection (32, 33). Another mechanism for protection against spike protein binding to epithelial cells is the use of decoy receptors. For example, other potential binding partners for SARS-CoV-2 spike protein at the epithelial surface are proteoglycans. Recent studies suggest a key role for proteoglycans in facilitating SARS-CoV-2 spike protein binding to the ACE-2 on the cell membrane (34, 35, 36). However, exogenous glycosaminoglycans, such as heparin, interfere with spike protein–epithelial proteoglycan interactions and may blunt viral epithelial binding and infection (37, 38). Alternatively, sACE-2 ectodomain binds to SARS-CoV-2, functioning as a decoy receptor to prevent infection of epithelial cells (39, 40, 41, 42). Recently, it was reported that recombinant human sACE-2 inhibits SARS-CoV-2 infections, supporting the efficacy of sACE-2 as a potential therapeutic to limit viral infection (17, 40). There is a current phase 2 clinical trial of inhaled rhACE-2 as a potential therapy for patients with COVID-19 (RhACE2 APN01, NCT04287686). We demonstrated that soluble ACE-2-Fc bound to SARS-CoV-2 spike protein with high affinity in a dose-dependent manner, so it is possible that sufficient concentrations of sACE-2 released by NE may interfere with viral–epithelial interactions.

Our study focused on the impact of NE cleavage of ACE-2 and its subsequent decreased activity as a receptor for SARS-CoV-2 spike protein. However, the sequelae of NE exposure on ACE-2 fate are not known. Using an *in vitro* cell culture system, Wang *et al.* (43) demonstrated that the time interval between loss of ACE-2 at the cell surface due to SARS-CoV-2 spike protein binding and internalization by an endocytic pathway revealed that reappearance of ACE-2 on the cell surface required about 14 h. In contrast to the endocytic pathway, it is possible that post-NE treatment, residual ACE-2 ectodomain remains on the cell membrane permitting SARS-CoV-2 spike protein binding albeit at reduced affinity (19). Another important consideration in studying the impact of NE on ACE-2–SARS-CoV-2 spike protein interactions is the potential confounding actions of other molecules on the epithelial surface or in the airway milieu of patients with CF that may influence SARS-CoV-2 spike protein binding to epithelial cells. For example, epithelial cell surface proteoglycans, heparan sulfate (35) or syndecans (44, 45) stabilize viral–epithelial interactions and promote infection. In contrast, in sputum from patients with CF, the presence of high concentrations of several anionic polymers such as DNA, mucins, and shed proteoglycans could potentially bind to the SARS-CoV-2 spike protein receptor-binding domain, a domain rich in positively charged amino acids, and interfere with spike protein binding to epithelial targets. Finally, although we did not test the impact of NE on SARS-CoV-2 infection of epithelial cells using an infection model, our results demonstrated a compelling association between NE cleavage of ACE-2 from the cell surface of primary HBE and significantly decreased SARS-CoV-2 spike protein binding to HBE cells. Failure of SARS-CoV-2 spike protein binding to epithelial cells is an important preclinical outcome measure for the potential to block SARS-CoV-2 infection (35, 46).

In summary, the evidence presented here indicates that NE decreased the binding of spike protein to HBE cells by reducing cellular ACE-2 expression *via* proteolytic shedding of the ectodomain. Furthermore, LC-MS/MS analysis established two candidate NE-ACE-2 cleavage sites and confirmed that NE cleaved the ACE-2 ectodomain in a region that contains the potential spike protein binding sites, providing at least a partial explanation for why there is less spike protein binding to HBE post-NE exposure. While there are studies on the cleavage of ACE-2 by ADAM17, little is known about NE-mediated ACE-2 shedding and its impact on coronavirus infection. Recent evidence demonstrated that recombinant sACE-2 could effectively interfere with binding of SARS-CoV-2 spike protein to its cellular receptor. Therefore, ACE-2 ectodomain release by NE could inhibit the SARS-CoV-2 virus infection by two mechanisms: (1) by reducing membrane-bound ACE-2 and (2) by increasing the abundance of sACE-2 protein in the airway milieu. These mechanisms may play a role in protecting people with CF from COVID-19 infections.

## Experimental procedures

### Sputum processing for ACE-2 quantitation and quantitation of plasma and sputum ACE-2 by ELISA

Sputa from patients with CF or mucus from endotracheal tubes of healthy adult patients and plasma from patients with CF or subjects with no CF were obtained following Virginia Commonwealth University (VCU) institutional review board approval and written consent in accordance with the “Declaration of Helsinki” and processed as previously described (47, 48). Briefly, sputum or mucus supernatants were prepared by the addition of normal saline (NS) containing 10% Sputolysin (catalog number 560000, Calbiochem, Millipore) (1:1, W: V), followed by incubation at 37 °C for 15 min. For testing correlation of sACE-2 levels with NE activity, sputum supernatants were prepared by the addition of NS-containing DNase-1 (0.3 mg/ml, catalog number DN25, Sigma-Aldrich) at the ratio of 1:1 (W: V), followed by incubation at 37 °C for 2 h. After incubation, sputum samples were centrifuged at 25,000*g* for 30 min at 4 °C to collect the sputum supernatant. Levels of sACE-2 were quantified in equal volumes of sputum supernatants and plasma samples, using sandwich ELISA kit following manufacturer’s instructions (Catalog number DY933-05, Human ACE-2 DuoSet ELISA, R&D Systems). Levels of sACE-2 were determined using standard curves constructed with recombinant ACE-2.

### Sputum processing for NE activity and NE assay

Processing of sputum samples for DNase-1 treatment, followed by measuring NE activity, was performed as described previously (49). Briefly, frozen sputum samples from CF subjects were thawed on ice, weighed, and mixed with NS alone or NS-containing DNase-1 (0.3 mg/ml) at the ratio of 1:1 (w: v). Diluted sputum samples were mixed by gentle vortex and incubated at 37 °C for 2 h. Following incubation, samples were centrifuged at 25,000*g* for 30 min at 4 °C to collect the sputum supernatant. The sputum supernatants were used to measure NE activity using a chromogenic 96-well micro titer plate assay with N-Methoxysuccinyl-Ala-Ala-pro-Val-p-nitroanilide (catalog number M4765, Sigma-Aldrich) as the substrate. First, purified human sputum NE was reconstituted in 1:1 glycerol:0.02 M sodium acetate (pH 5) buffer and included in the assay to create a standard curve, ranging from 0.23 to 30 μg/ml (0.01–1.0 μM) in 125 mM Hepes buffer (pH 7.5, 0.125% Triton-X 100). Sputum supernatants were diluted 1:1 (100 μl) in Hepes buffer, loaded on a micro titer plate, incubated at room temperature (RT) for 15 min with gentle agitation, and then, the reaction was initiated by the addition of the substrate (3 mM in 50% dimethyl sulfoxide, 50 μl), specific chromogenic substrate for NE. The rate of hydrolysis of the substrate was measured at 405 nm and recorded at 30 s intervals for 5 min. NE activity was calculated from the slope of the time-dependent increase in *A* at λ = 405 nm using the NE standard curve.

### Submerged and ALI cultures of HBE cells

The protocol was approved by the Institutional Review Board for clinical investigations, Duke University Medical Center to use deidentified human lung donor airway tissue discarded during lung transplant procedure. Primary HBE cells were harvested and cultured as previously described (50, 51). Briefly, human tracheobronchial tissues obtained from the Lung Transplant Program in the Department of Pathology at Duke University Medical Center were processed by proteolytic digestion (Protease Type XIV, Sigma). Cells from different donors were harvested. After protease treatment, NHBE cells harvested from different donor tissues were included in the study. Initial plating and expansion was performed on PureCol- (catalog number 5005-B, Advanced BioMatrix) coated 10-cm tissue culture dishes in small airway basal media (Lonza):DMEM-H (1:1) supplemented with seven factors: insulin (4 μg/ml), transferrin (5 μg/ml), epidermal growth factor (0.5 ng/ml), dexamethasone (0.1 μM), cholera toxin (20 ng/ml), bovine pituitary extract (50 mg/500 ml), and bovine serum albumin (0.5 mg/ml). After initial harvest and expansion, cells were either culture-submerged for obtaining undifferentiated HBE cells or seeded on a six-well Trans well inserts (0.4 μM, 24-mm diameter, Costar) and cultured in ALI in serum-free and growth factor supplemented medium for obtaining differentiated HBE cells as previously described (52). Cells were cultured in ALI for 10 to 12 days before experiments.

### HBE cells and NE treatment

HBE cells, with undifferentiated phenotype culture-submerged on 60 mm dishes or with differentiated phenotype cultured for 10 to 12 days at ALI, were washed twice with cold PBS and stimulated with NE (200 or 500 nM, catalog number SE563, Elastin Products) or vehicle control in a serum- and growth factor–free media and incubated in a humidified CO_2_ incubator for 1 to 2 h. For ALI cultures, cells were treated on both apical and basolateral surfaces. At the end of each experiment, NE activity was stopped with NE-specific inhibitor (MeOSuc-Ala-Ala-Pro-Val Chloromethyl Ketone [AAPV CMK, 10 μM], catalog number M0398, Sigma). Following treatments, conditioned media was evaluated for ACE-2 ectodomain fragment release by Western blot analysis. To enrich the secreted protein concentrations in the conditioned media, the samples were concentrated by 10-fold using Amicon ultra centrifugal filters (catalog number UFC500396, Ultracel, 3K, Millipore) and the concentrated conditioned media was stored at −80 °C until further use. For ALI cultures, conditioned media obtained from apical side showed sACE-2 expression while basolateral media with minimal detection.

### Western blot analysis for sACE-2 in conditioned media

Levels of cleaved ACE-2 fragment release in the conditioned media obtained from HBE cells were determined using Western blot analysis. Equal amounts of sample of concentrated conditioned media (22 μl) were separated on a 4 to 20% SDS-PAGE gradient gel, and proteins were electrotransferred onto a nitrocellulose membrane. Nonspecific binding sites were blocked with 5% milk in TBS-T buffer (20 mM Tris–HCl, 150 mM NaCl, and 0.1% Tween-20, pH 7.6) for 1 h at RT. The membranes were incubated with primary rabbit mAb against ACE-2 (1:1000, catalog number ab108252, Abcam) overnight at 4 °C. After washing with TBS-T, the membrane was incubated for 1 h at RT with a horseradish peroxidase–labeled anti-rabbit immunoglobulin G (IgG) (1:4000, catalog number 7074, CST). After washing, immunoreactive protein complexes were detected using SuperSignal Chemiluminescent Substrate (catalog number 34580, Thermo Fisher Scientific). (Catalog number NEL112001EA, PerkinElmer).

### Flow cytometry analysis of ACE-2-spike protein binding

To determine the SARS-CoV-2 spike protein binding to its cellular ACE-2, undifferentiated primary HBE cells, harvested with an enzyme-free cell dissociation buffer (catalog number 13151014, Thermo Fisher Scientific), were incubated with recombinant SARS-CoV-2 trimeric spike protein with C-terminal His6-tag (catalog number SPN-C52H8, Acro BIOSYSTEMS) (1, 5, or 10 μg/ml) for 90 min at 37 °C. His6 peptide (catalog number A6006, ApexBio) with matching concentrations and secondary antibody alone were included as controls. To measure the binding of His-tagged spike protein to the cell surface, the cells were stained with rabbit anti-His-tag antibody (1:500, catalog number 12698, CST) for 1 h at RT, followed by Alexa Fluor 488–conjugated goat anti-rabbit IgG (1:500, catalog number 4412, CST) for 30 min. To determine NE effect on spike protein binding, HBE cells were treated with NE (200 or 500 nM) or vehicle control for 2 h prior to incubation with SARS-CoV-2 spike protein (10 μg/ml). Cell-associated fluorescence was measured on a BD LSRFortessa-X20 analyzer (Becton-Dickinson), data was acquired with BD FACSDiva (version 9; Becton-Dickinson, https://www.bdbiosciences.com/en-us/products/software/instrument-software/bd-facsdiva-software), and data was analyzed with Flow Jo software (version10.8.0; Becton-Dickinson, https://www.flowjo.com/solutions/flowjo). Positively stained cells had fluorescence measurements above the background fluorescence of the isotype control. DAPI stain was added to samples just prior to acquiring on the flow cytometer, to ensure only live cells were assessed. Live cells were defined as negative for DAPI staining. The MFI of at least 50,000 live cells were recorded and used for analysis for each sample. For the negative control, primary antibody was omitted.

### *In vitro* cleavage assay

Direct cleavage of ACE-2 and SARS-CoV-2 spike protein by NE were tested *in vitro* using recombinant human ACE-2 protein with Fc-tag at the C terminus (catalog number AC2-H5257, Acro BIOSYSTEMS) or SARS-CoV-2 spike protein, His tag, active trimer (catalog number SPN-C52H8, Acro BIOSYSTEM). Purified ACE-2 (750 ng) or SARS-CoV-2 spike protein (750 ng) was incubated in a reaction buffer (10 mM Hepes, pH 6.0, 1 mM DTT) with NE (50, 100, or 200 nM; 15 or 30 min, 37 °C), and reaction products were separated by 4 to 20% SDS PAGE. The proteolytic products were detected by staining the gels with imperial protein stain (catalog number 24615, Thermo Fisher Scientific), and the fragments of ACE-2 and the Fc-tag were further confirmed by Western analyses using rabbit monoclonal anti-ACE-2, that recognizes the ectodomain of the protein,(1:1000, catalog number ab108252, Abcam) and mouse mAb raised against human IgG_1_ Fc region (1:1000, catalog number MAB110, R&D), followed by goat-anti-rabbit or mouse-horseradish peroxidase–conjugated antibody and immunoreactive protein complex detection with SuperSignal West Pico Chemiluminescent Substrate (Thermo Fisher Scientific).

### Flow cytometry analysis for cell surface ACE-2 expression

To measure the ACE-2 expression at the cell surface, HBE cells grown in submerged culture were harvested and treated with NE (200 and 500 nM) or control vehicle in suspension for 2 h before incubation with goat anti-human ACE-2 (1 μg/reaction, catalog number AF933, R&D) antibody that recognizes the extracellular domain of ACE-2, rocking for 2 h at RT. Following incubation, cells were washed and further incubated with PE-conjugated donkey-anti goat IgG (10 μl/reaction, catalog number F0107, R&D), rocking at RT for 60 min. The cell surface expression of ACE-2 was determined by measuring the PE fluorescence intensity on a flow cytometer (LRSFortessa-X20 analyzer, Becton-Dickinson), and the data was analyzed with Flow Jo software. Live cells were gated as a DAPI negative population, and the MFI of at least 50,000 cells were recorded and used for analysis of each sample. Control conditions included were auto fluorescence (absence of primary and secondary antibody) and PE-conjugated secondary antibody only.

### In-gel digestion and peptide extraction

The in-gel digestion protocol was performed as described (25). Following in-gel reduction and alkylation, the gel bands were incubated overnight at 37 °C in a 100 μl solution consisting of 20 ng/μl trypsin and 90:10 (v: v) 10 mM ammonium bicarbonate (pH = 7.8):acetonitrile. Peptides were extracted in 100 μl of a 1:2 (v: v) 5% formic acid:acetonitrile solution for 15 min at 37 °C followed by drying the solution in a speedvac and storage at −20 °C.

### Liquid chromatography tandem mass spectrometry

The in-gel digest samples were analyzed by LC-MS/MS using a Thermo Fusion Lumos coupled to a Thermo Easy-nLC system operated in a data-dependent acquisition mode. The Fusion Lumos source was set to 1900 V (pos) and the ion transfer temperature maintained at 275 °C. Full scan = 400 to 1800 *m/z* at an automatic gain control (AGC) = 2E6, IT_max_ = 50 msec, μscan = 1, and resolving power = 120 K. MS/MS were generated at an isolation width = 1.6 *m/z*; stepped high-energy collision dissociation = 15, 30, 45 normalized collision energy; AGC = 1E5; ITmax = 54 msec; μscan = 1; and resolving power = 30 K. The data-dependent acquisition cycle consisted of a full scan mass spectrometry (MS) followed by MS/MS scans for 3 s with the dynamic exclusion = 60 s. Dried in-gel digestion samples were reconstituted in 50 μl of 0.1% formic acid and injected (1 μl) onto a 0.75 × 250 mm Aurora 1.6 μm C18 reverse phase ultra-performance liquid chromatography (UPLC) column (IonOpticks) housed in a column oven set to 40 °C. Each sample was eluted at 300 nl/min over a 70 min gradient as follows: 0 to 40 min (3–35% B), 40 to 50 min (35–100% B), 50 to 60 min (100% B), 60 to 65 min (100–3% B), and 65 to 70 min (3% B). Mobile phase compositions were as follows: A = 0.1% formic acid and B = 0.1% water:acetonitrile (2:98). Proteomic datasets were processed in Proteome Discoverer 2.5 software (https://www.thermofisher.com/us/en/home/industrial/mass-spectrometry/liquid-chromatography-mass-spectrometry-lc-ms/lc-ms-software/multi-omics-data-analysis/proteome-discoverer-software.html) and searched with SEQUEST HT against the recombinant ACE-2-Fc FASTA sequence and SwissProt *Homo sapiens* proteome database (download: 6/11/2021). LC-MS/MS data were searched using the following critical conditions: protease = neutrophil elastase + trypsin; full scan MS mass accuracy = 10 ppm; MS/MS = 0.02 Da; minimum peptide length = 4 amino acids, fixed modifications = carbamidomethyl (Cys); variable modifications = acetyl (N-term) and oxidation (Met); and a false discovery rate of 1%. Label-free quantitative values included the summed abundances of all peptides, normalized to the total peptide amount, missing values not imputed, and log_2_ transformed for quantitative analysis.

### Statistical analysis

Data were expressed as mean ± SEM of biological replicates of independent experiments. Statistical analyses were performed using Statistix 8.0 (Analytical Software, *Statistix 8*; www.statistix.com). For comparison of three or more unmatched treatment groups, a one-way, nonparametric ANOVA (Kruskal–Wallis) test, followed by post hoc comparisons using the Wilcoxon rank sum test, were used to determine significant differences between treatment groups. For comparison of two unpaired groups, especially ACE-2 levels in sputum and plasma samples, an unpaired two-tailed *t* test with a Mann-Whitney U was used. Concentrations of ACE-2 and NE activity levels in CF sputum were log transformed to normalize their distribution, and linear regression was used to assess the association between ACE-2 and NE activity levels using GraphPad Prism 5 (GraphPad software, Inc; https://www.graphpad.com/). Differences between groups were considered significant at *p* < 0.05.

## Data availability

All representative data contained within the article including the supporting information are available upon request.

## Supporting information

This article contains supporting information (6).

## Conflict of interest

The authors declare that they have no conflicts of interest with the contents of this article.
