# Supplementary material for: Neutrophil elastase decreases SARS-CoV-2 spike protein binding to human bronchial epithelia by clipping ACE-2 ectodomain from the epithelial surface
Source: J Biol Chem. 2023 May 13;299(6):104820. doi: 10.1016/j.jbc.2023.104820 (PMC10181948; doi:10.1016/j.jbc.2023.104820)
Supplement: Supporting information [file mmc1.docx]

**Supporting Information**

**Neutrophil Elastase decreases SARS-CoV-2 Spike protein binding to human bronchial epithelia by clipping ACE-2 ectodomain from the epithelial surface**

Apparao B. Kummarapurugu^1*^, Adam Hawkridge^2^, Jonathan Ma^1^, Stephanie Osei^3^, Rebecca K. Martin^4^, Shuo Zheng^1^, Judith A. Voynow^1^.

^1^Department of Pediatric Pulmonary Medicine, Children’s Hospital of Richmond at Virginia Commonwealth University, Richmond VA, ^2^School of Pharmacy at Virginia Commonwealth University, Richmond VA., ^3^Virginia Commonwealth University, Richmond VA, ^4^Department of Microbiology and Immunology at Virginia Commonwealth University, Richmond VA.

**Supplementary Methods**

Protein-Protein Pull-Down Assay: To determine ACE-2 receptor ectodomain binding to SARS-CoV-2 spike protein, protein pull-down assay was performed as previously described (1). Briefly, carboxy-terminal His-tagged recombinant SARS-CoV-2 spike protein (2 µg) was immobilized by incubating with dynabeads (40 µL) (Catalog number 10103D, Invitrogen), specific for his-tag protein in binding/wash buffer (300mM NaCl, 2mM KH_2_Po_4_, 6mM Na_2_HPO_4_, 0.01% Tween 20 and protease inhibitors), 20 min at RT with rocking. Following incubation, spike protein bound dynabeads were washed and equal amounts of spike protein coupled beads prepared in pull-down buffer (77mM NaCl, 1mM KH2PO4, 3mM Na2HPO4, 0.01% Tween 20 and protease inhibitors), were mixed with different concentrations of carboxy-terminal Fc-tagged recombinant ACE-2 protein (0-160ng) and incubated for 2h at RT with rocking. Following incubation, dynabeads with bound protein complexes were washed 3 times. Bead-associated protein complexes were eluted with SDS gel loading buffer and resolved on 4-20% SDS-PAGE under reducing conditions. The protein complexes were identified using anti-ACE-2 antibody for ACE-2 or anti-His tag antibody for spike protein by Western blot analysis as described in the experimental procedures section.

**Supplementary Results**


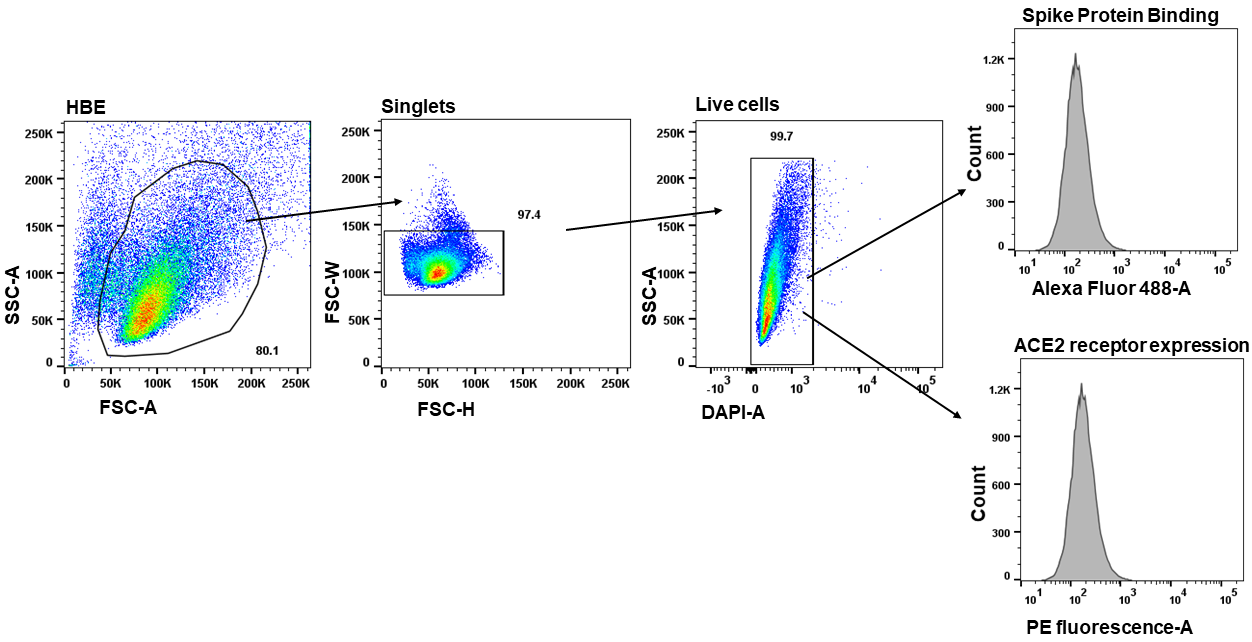
Figure S1. A representative schematic showing the flow cytometry gating strategy for measuring cell surface ACE-2 receptor expression (Fig.3), and spike protein binding to ACE-2 receptor (Fig.4) in HBE cells. Single cells, negative for DAPI staining were considered live**.**

**Recombinant Fc-tagged ACE-2 receptor bound to SARS-CoV-2 trimeric His6-tagged spike protein with high affinity *in vitro***

We sought to evaluate the binding strength of the extracellular domain of recombinant Fc-tagged ACE-2 with SARS-CoV-2 trimeric His6-tagged spike protein in vitro using a protein pulldown assay. To study the affinity of interaction, a fixed quantity of His6-tagged recombinant SARS-CoV-2 spike protein, immobilized to Cobalt-coated magnetic dynabeads, was incubated with different concentrations of carboxy-terminal Fc-tagged recombinant ACE-2 protein (0-160ng). Following incubation, the complexes that were pulled down with the dynabeads were evaluated by Western analysis for ACE-2. There was dose-dependent binding of ACE-2 (upper panel) with spike protein (**Fig. S2**). To confirm that pull-down was due to spike protein immobilized to Dynabeads, western analysis of the input samples confirmed the presence of intact spike protein with the molecular weight of ~150kDa (lower panel).

Figure S2. Protein pull-down assay to detect interaction of ACE-2 receptor ectodomain and SARS-CoV-2 spike protein. Recombinant SARS-CoV-2 spike protein with His tag immobilized on Ni-coated magnetic dynabeads were incubated with Fc-ACE-2 protein (5-160 ng, 90 min, RT) in pull-down buffer. Dynabeads conjugated with SARS-CoV-2 spike protein without incubation of Fc-ACE-2 protein served as control. Following incubation, the beads were pelleted, washed and eluted in 1x SDS-PAGE sample buffer. Equal volumes of eluate were separated by 4-20% SDS-PAGE electrophoresis. Following transfer, the blots were probed with human anti-ACE-2 to detect ACE-2 protein (upper panel) or anti-His-Tag antibody to detect SARS-CoV-2 spike protein (lower panel). Data shown is representative of two independent experiments. PC, positive control.


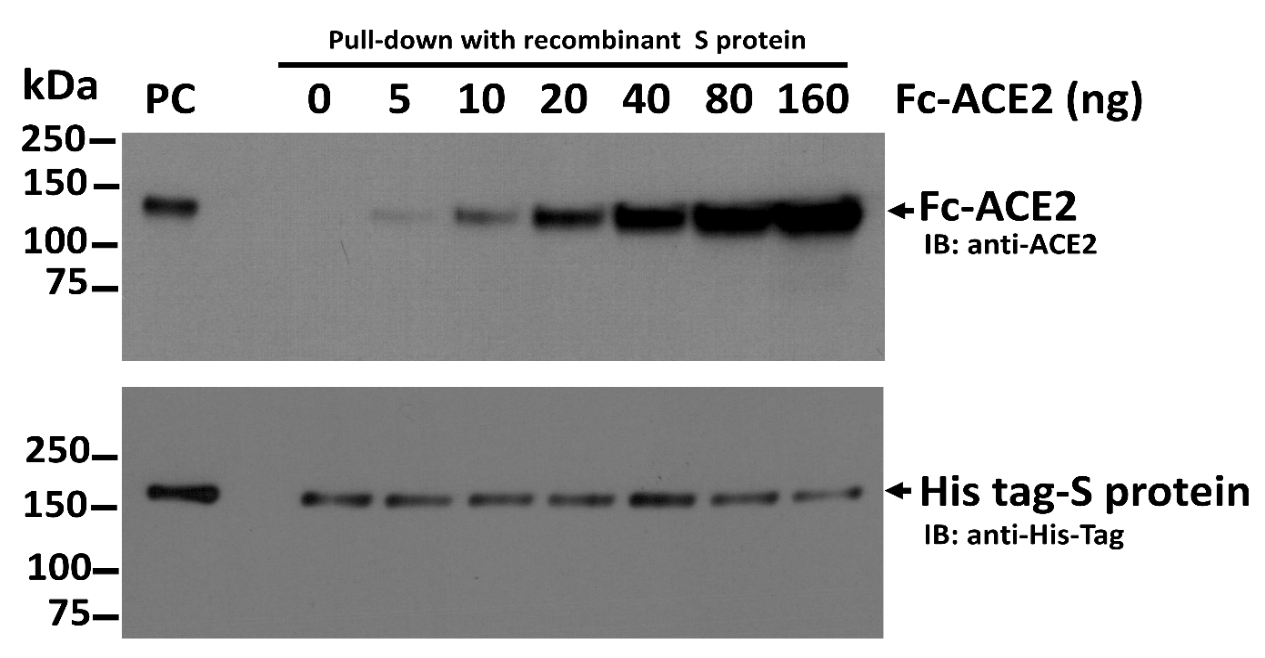


Figure S3. Assessment of cell surface ACE-2 expression in HBE cells by flow cytometry. Undifferentiated HBE cells expressing endogenous ACE-2 were exposed to NE (200 or 500nM) or vehicle control at 37°C for 2h. Following incubation, the cells were stained with goat anti-ACE-2 antibody (1µg/ml) followed by phycoerythrin conjugated donkey anti-goat IgG (1:100). Cell surface ACE-2 expression in HBE cells following NE treatments was analyzed by flow cytometry and summarized in Fig.3 Individual flow cytometry histograms presented here showing the decreased ACE-2 staining following exposure of NE 200 (green) or 500nM (blue) or vehicle control (black) for all three biological experiments, with replicates, using different HBE donor cells. Background staining was determined for HBE cells exposed to secondary antibody only control (red bar).


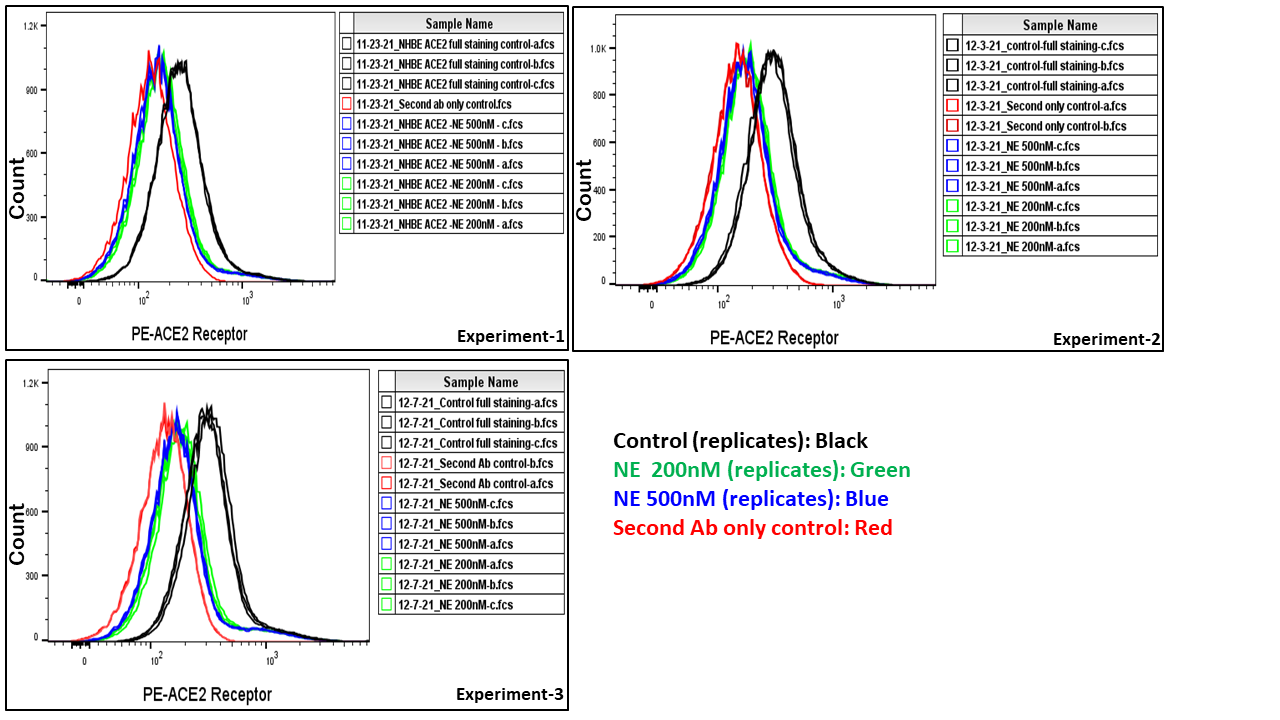


Figure S4. In-vitro cleavage of recombinant ACE-2-Fc tagged protein by NE. Recombinant human ACE-2 protein with Fc tag was incubated with NE (0, 50 or 100nM) for 30 min and equal amount of reaction products were resolved on 4-20% SDS-PAGE. Dose dependent cleavage ACE-2-Fc tagged protein by NE was determined by densitometry of protein bands following Imperial Protein Stain, and results are presented as a percentage of the control (0 nM NE). Results are presented graphically (mean + SE), n=4 independent experiments with a total of 5 replicates per condition.


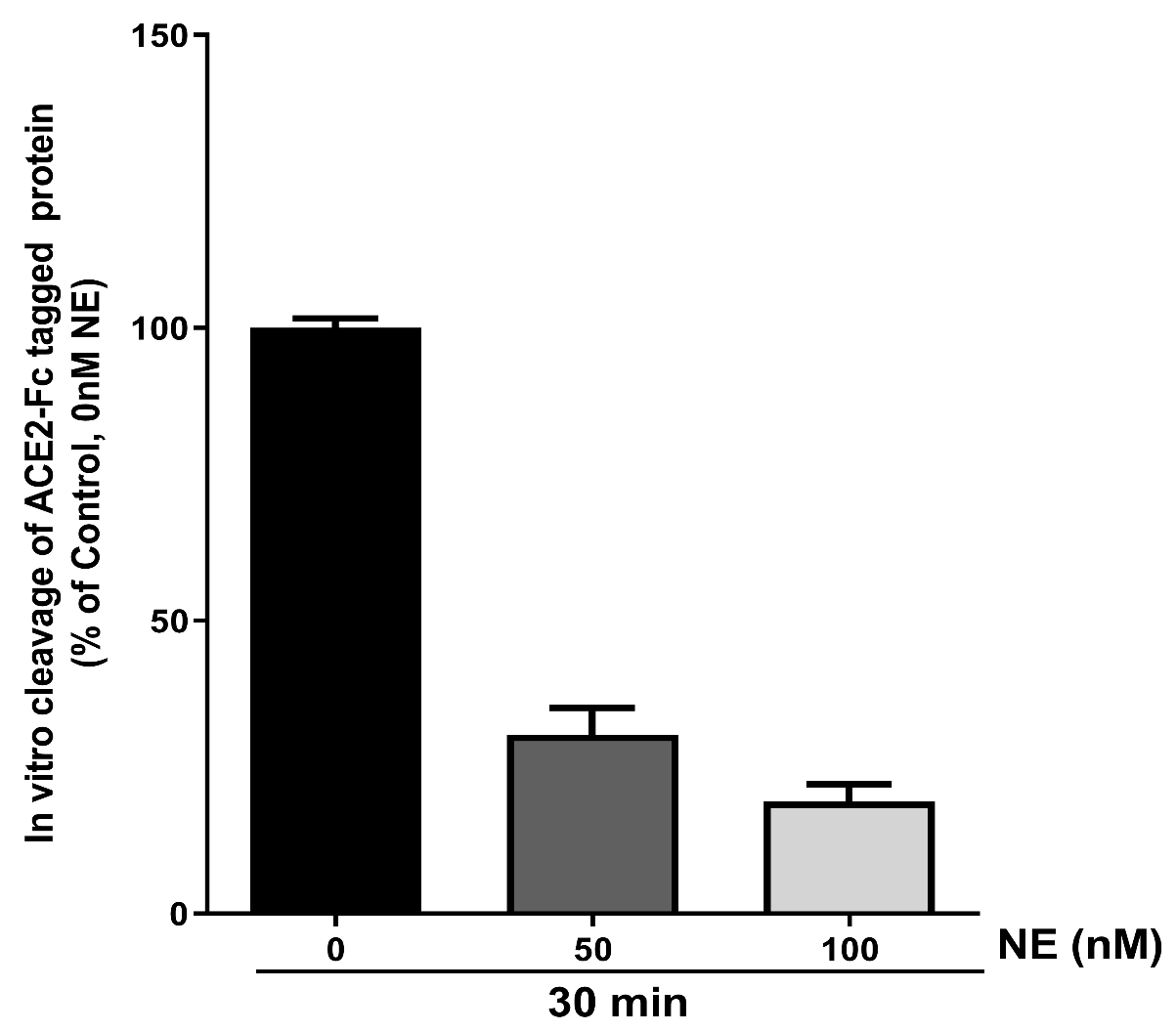


**Supplementary references**

1. Shang, J., Wan, Y., Luo, C., Ye, G., Geng, Q., Auerbach, A., and Li, F. (2020) Cell entry mechanisms of SARS-CoV-2. *Proc Natl Acad Sci U S A* **117**, 11727-11734.
